# Effects of interventional public health laws and regulations intended to reduce gambling-related harms: a realist review study protocol

**DOI:** 10.1136/bmjopen-2024-093906

**Published:** 2025-07-16

**Authors:** Michelle Fisher, Theodore Piper, Sonia Mavi, Jaya Nambiar, Priyanka Kumar Sharma, Joelle Kirby, G J Melendez-Torres, Paul Montgomery, Graham Fewell, Joht Singh Chandan, Kate Bedford

**Affiliations:** 1 University of Birmingham College of Medicine and Health, Birmingham, UK; 2 University of Birmingham, Birmingham, UK; 3 University of Exeter, Exeter, UK; 4 Department of Social Policy, Sociology and Criminology, University of Birmingham, Birmingham, UK

**Keywords:** Systematic Review, Legislation, PUBLIC HEALTH

## Abstract

**Abstract:**

**Introduction:**

Gambling is now widely acknowledged to be a major public health (PH) issue. The Office for Health Improvement and Disparities conservatively estimated that gambling harm is associated with an annual cost of £1.05–£1.77 billion in England alone. Marionneau *et al* have categorised gambling harms into seven themes: (1) financial, (2) relationship/conflict, (3) emotional and psychological (mental health), (4) health decrements (physical health), (5) employment/education, (6) cultural and (7) criminal activity. In this understanding, gambling harms are not restricted to individual experiences: they also impact families, the wider community and society, and hence they require a whole systems, PH approach, anchored in population-level interventions to reduce harms. We aim to identify the effects of interventional PH laws and regulations on the harms associated with gambling.

**Methods and analysis:**

We limit our focus to interventional PH laws and regulations within a comprehensive search of scientific and legal databases, grey literature and books. Following Population, Intervention, Comparator, Outcome, Study, Timing inclusion criteria, evidence will be screened and appraised in Covidence by two reviewers (MF and TP). Included evidence will be analysed and synthesised using a narrative synthesis approach. Methodological quality will be appraised using the relevant risk of bias tool. Randomised controlled trials will be assessed using the Cochrane risk of bias tool (RoB2), Risk Of Bias In Non-randomised Studies - of Interventions (ROBINS-I) will be used for other non-randomised studies. Qualitative studies will be appraised using the EPPI reviewer software for systematic reviewing.

**Ethics and dissemination:**

The review protocol is registered with PROSPERO (International prospective register of systematic reviews) at the National Institute for Health Research and the Centre for Reviews and Dissemination (CRD) at the University of York (CRD42024574502). We aim to define a theory of change and produce a context-mechanism-outcome framework with relevant experts using the findings. We plan to disseminate the findings through peer-reviewed publications, meetings with relevant experts and international conference presentations.

**Strengths and limitations of this study**

- We aim to include a far wider range of evidence than usually assessed within health research.
- Industry and non-industry funded data sources will be screened separately.
- We aim to facilitate a rigorous interdisciplinary knowledge translation approach.
- To our knowledge, no reporting guidance (eg, Preferred Reporting Items for Systematic Reviews and Meta-Analyses) exists for reviewing legal interventions in health research.

## Introduction

### Rationale

 Gambling is now widely acknowledged to be a major public health (PH) issue. A 2021 Public Health England evidence review^1^ and a subsequent 2023 Office for Health Improvement and Disparities (OHID) update^2^ found substantial morbidity and mortality associated with gambling. While noting the lack of comprehensive data on gambling harms, by using evidence on costs of suicide, depression, unemployment, imprisonment and homelessness, OHID conservatively estimated that gambling harm was associated with an annual cost of £1.05–£1.77 billion in England alone. The National Institute of Economic and Social Research estimates problematic gambling to cost £1.4 billion per year. In response, many experts call for a whole systems, PH approach, anchored in population-level interventions to reduce harms.

Early gambling harms research was mainly focused on individual ‘problem gamblers’, and screening models maintain a focus on individuals.^13^,^5^ However, more recent research has incorporated wider dimensions of harm, including at the population level. For example, in line with other work adopting a PH perspective, Marionneau *et al* have categorised gambling harms into seven themes: (1) financial, (2) relationship/conflict, (3) emotional and psychological (mental health), (4) health decrements (physical health), (5) employment/education, (6) cultural and (7) criminal activity.^3 6^ In this understanding, gambling harms are not restricted to individual experiences: they also impact families, the wider community and society.^7^,^9^

Experts who advocate for a PH approach to gambling regularly call for legal and regulatory interventions.^10^,^12^ These interventions can include prohibition, age restrictions on access to products or premises, advertising and marketing limits, bans on particular types of machines or games, stake limits and restrictions on the physical and temporal availability of gambling. Legal epidemiological responses have included significant legal and regulatory changes, underpinned by concerns about harm, which have been enacted in recent years (eg, changing the age limit for the National Lottery; strengthening the Licensing Conditions and Codes of Practice for remote operators).^13 14^ Further measures were proposed in the 2023 White Paper on gambling, with legal changes subsequently introduced around stake limits on online slots, mandatory affordability checks for online gamblers when their spending reaches a certain threshold, and lower online slot stake limits for 18–24 years.^15^ Further, the Gambling Commission’s 2023–2026 plan^2^ to improve the evidence base for gambling regulation repeatedly identifies the urgent need to assess which interventions are most effective in reducing gambling-related harms, and to improve the use of evaluative approaches. Legal interventions are a crucial focus.

As described in legal epidemiology, a wide variety of laws and regulations have incidental impacts on PH,^16^ but as noted by Burris *et al*^17^ the key subset of ‘interventional public health law’ consists of ‘law or legal practices that are intended to influence health outcomes or mediators directly’.^17^ While such interventional law can be a powerful PH tool, we know from other sectors that it can sometimes not work as intended, and it can often have differentiated effects on various groups within a jurisdiction. Research outside gambling confirms that interventional PH laws and regulations need to be evaluated for effectiveness,^18^ including for their impact on inequalities.^17 19^ Key questions include whether law can be empirically shown to have an impact on the health of the population, and whether it is possible to identify the ways in which law affects health inequalities. Furthermore, attention to unintended effects of interventions is standard within regulatory impact assessments,^20^ and studies of intervention-generated inequalities routinely consider differences in intervention effect between different groups.^1921^,^24^ PH law scholars have directed particular attention to focusing on the needs of the most vulnerable in this regard, such that justice is understood as both ‘health improvement for the population and fair treatment of the disadvantaged’.^25 26^ The aim is to ensure that efforts to address health inequalities are equitable, including by considering the impacts of interventions on disadvantaged subgroups.^17 27 28^ To craft effective interventional PH laws and regulations, then, researchers need to take a legal epidemiological approach to scientifically review relevant evidence about what works, for whom and what the positive and negative effects^29^ of specific laws and regulations intended to directly influence health outcomes may be.

However, there is no systematic, comprehensive, up-to-date review of evidence on the effectiveness of legal interventions intended to address gambling harm.^30^ The Lancet Public Health Commission on gambling has gathered descriptive information about trends in gambling legislation in different countries,^31 32^ but there is a paucity of work systematically examining the effects, including the health equity effects, of laws and regulations intended to reduce gambling harm. Many studies of intervention effectiveness bundle legal and regulatory measures together with behavioural, treatment, educational or policy interventions, meaning separate analyses of legal interventions cannot easily be conducted.^15 33^ A 2019 mapping review of interventions addressing gambling-related harms searched only in English; only from 2012; and only in medical and limited social sciences databases.^34^ While including studies on novel meditative practices and pharmaceutical interventions, it missed key research on legal and regulatory interventions aiming to reduce harm, such as on the potentially counter-productive impacts of cashless play rules on older people (who use cash to limit spending).^35^,^41^ In addition, since 2019, there have been numerous additional studies of legal interventions to reduce harms related to gambling,^42^,^44^ including in law journals.^40 42 45 46^ In short, there is a rich, interdisciplinary evidence base about the effects of laws and regulations intended to reduce gambling harm, but it needs to be systematically gathered, evaluated and synthesised, and the causal mechanisms explaining outcomes need to be explicated by a team with combined sociolegal and PH expertise.

**Figure 1:**
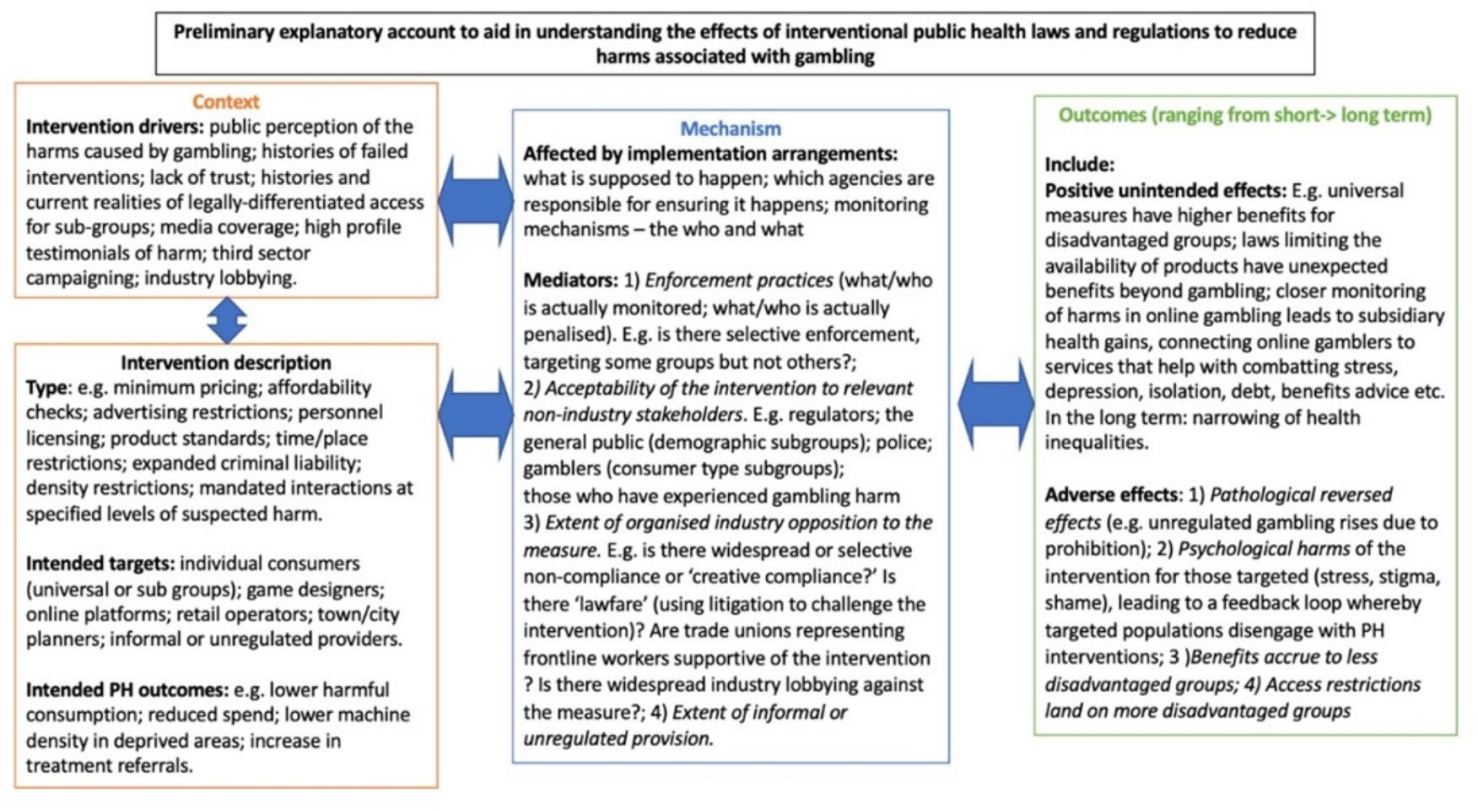
Draft CMOC codesigned with PPI. CMOC, context-mechanism-outcome configuration; PPI, patient and public involvement.

As a first step, with patient and public involvement (PPI) input, we generated a preliminary explanatory context-mechanism-outcome configuration (CMOC) model of pathways for unintended interventional effects of laws intended to reduce gambling harms (see figure 1 below). This will guide the focus of our systematic review, incorporating factors important to patients and the wider public. The CMOC also provides a framework for synthesis to be adapted and expanded.

#### What we propose to do

Specifically, to craft effective interventional PH laws and regulations to address gambling harm, lawmakers need researchers to review relevant evidence about what works, for whom, and what the positive and negative effects^29^ of specific laws and regulations may be. Negative effects include paradoxical effects (eg, interventions aiming to reduce gambling that appear to increase it), and harmful externalities (eg, psychological harms or equity harms).^29 47^ The methods used to measure intervention effectiveness must be comprehensive, drawing on diverse bodies of evidence, including diverse stakeholder perspectives, and accounting for the breadth of intended/unintended and spillover effects to capture their impact.^48^,^50^ In so doing, we can improve our understanding of intervention efficacy and mechanisms for efficacy and identify lessons for how future interventions should be designed.

### Research questions

1. What are the effects of interventional PH laws and regulations intended to reduce harms associated with gambling on any global population or subpopulation?
2. What are the mechanisms by which those laws result in their outcomes?
3. What are the contexts that influence those mechanisms?

### Objectives

1. Identify the effects of interventional PH laws and regulations intended to address gambling harm.
2. Conduct a realist synthesis to provide an initial explanatory analysis of how and why legal and regulatory interventions work (or do not work) in particular contexts or settings, for particular subgroups.^51^,^53^
3. Share that initial explanatory analysis (derived from the systematic review evidence) with a range of experts, to coproduce a second iteration, codesigned CMOC.

## Methods

This review will explore all effects of interventional PH laws and regulations intended to address gambling harms. The reporting of the protocol follows the Preferred Reporting Items for Systematic Review and Meta-Analysis Protocols (PRISMA-P) guidelines.^54^

### Patient and public involvement

We have involved members of the public in the design of our realist review protocol and explanatory CMOC framework. GF and other lived experience partners provided feedback on the manuscript and were included in the coproduction of a framework describing pathways of unintended harm from gambling harm reduction regulation and laws.

### Eligibility criteria

In this review, we limit our focus to interventional PH laws and regulations ‘intended to improve the health of a defined population through specific preventive interventions’.^17 55^ They consist of primary and secondary legislation (including statutory instruments), and related regulations, ordinances, licensing conditions and codes of practice and binding guidance. As noted by the UK government, the key factor distinguishing legal and regulatory interventions from other types of rules (eg, within voluntary industry schemes) is whether ‘failure to comply would result in the regulated entity or person coming into conflict with the law or being ineligible for continued funding, grants and other applied-for schemes’.^20^ We do not consider case law in this study. We include only interventional PH laws and regulations intended to reduce gambling harms. Hence, we do not include smoking bans, even though there are many studies of the impacts of such bans on gambling premises, because smoking bans were not intended to reduce gambling harm. We would include fiscal measures designed to address gambling harms (eg, a statutory levy to fund expanded treatment), but not a general shift in gambling duty unrelated to PH and intended merely to raise revenue.

A comprehensive search is particularly important as previous efforts to synthesise the evidence in this space have been limited by non-inclusion of important multidisciplinary sources. This may have underestimated the impacts and/or inequalities generated through interventional PH laws and regulation.

Inclusion criteria follow the Population, Intervention, Comparator, Outcome, Study design, Timing (PICOST) format (see table 1).

**Table 1:** PICOST table

Subject	Concept
Population	Any study describing any global human population or subpopulation.
Intervention	Interventional PH laws and regulations intended to reduce the harms associated with gambling.Interventions can be single component or part of a multicomponent package, as well as universal, selective (eg, access restrictions targeting young adults) or indicated (eg, mandated affordability monitoring informed by demographic and individual risk profiles).
Comparator	None
Outcome	Health and health equity outcomes, for gamblers and affected others (eg, family, friends). For example: Reduced Problem Gambling Severity Index score. Reduced gambling spend and/or reported financial harm. Mental distress, depression, anxiety and negative emotional consequences. Negative behavioural consequences including criminal behaviour. Relationship happiness, relationship assessment. Outcomes by subgroups in the general population (PROGRESS-PLUS categories^63^) to allow us to assess for any inequity-generated impacts of a legal/regulatory intervention within a jurisdiction. Measures of compliance and enforcement. Cost-effectiveness. Positive, negative and spillover effects (including economic costs, paradoxical effects and harmful externalities, including widened health inequalities).
Study design	Randomised and non-randomised controlled trials. Prospective and retrospective cohort studies. Controlled before-and-after studies, including econometric studies, interrupted time series studies and regulatory impact studies. Observational studies, meta-analyses, modelling studies. Impact, process and economic evaluations (both cost–benefit analysis and cost-effectiveness analysis). Studies that seek to relate intervention costs and savings to health and well-being outcomes or benefits. Studies of moral hazard (a key law and economics framework for discussing unintended consequences). Any qualitative studies of the health equity effects of relevant legal interventions.
Timing	Date of publication. Timeframe of measurement of intervention. Date on which law/regulation came into effect. Length of time between enactment of law/regulation and measurement of intervention effect.

PH, public health; PICOST, Population, Intervention, Comparator, Outcome, Study design, Timing.

#### Inclusion criteria

1. Any study on the effects of a PH legal intervention which intends to reduce gambling-related harm.
2. Studies originating from any country and in any language.
3. Any study which reports any gambling-related harm/s as an outcome (defined using a seven-category, PH-related definition of such harms).^3 7^
4. Industry and non-industry funded research. (Industry funded research will be screened separately and act as a comparison group).

#### Exclusion criteria

1. Studies which do not include a PH legal intervention specifically intended to reduce gambling-related harm.
2. Studies which do not report outcomes related to gambling-related harm.
3. Case law.

### Information sources

We aim to include a far wider range of evidence than usually assessed within health research (eg, including law/sociolegal studies; history; politics; sociology and anthropology). Accordingly, we plan to implement searches of the following databases:

- CDC’s Task Force on Community Preventive Services (Community Guide).
- Health Evidence database (Canada).
- MEDLINE, Embase, CINAHL, PsycINFO, Social Policy and Practice (OvidSP).
- Cochrane Database of Systematic Reviews (CDSR) and the Cochrane Central Register of Controlled Trials (CENTRAL).
- NHS Economic Evaluation Database (via the Centre for Reviews and Dissemination).
- Social Science Citation Index and Conference Proceedings Citation Index (Web of Science, Clarivate Analytics); EconLit, Criminal Justice Abstracts (EBSCOhost); Lexis Nexis; Westlaw; Heinonline; Scopus.
- ProQuest Dissertations & Theses Global, Sociological Abstracts including Social Services Abstracts (ProQuest).
- Trials Register of Promoting Health Interventions (TRoPHI) and Bibliomap (EPPI-Centre).
- Campbell Systematic Reviews (Campbell Collaboration).

Important data for our purposes also exists in grey literature, especially regulatory impact assessments, which use templates and calculators to assess impact, and postimplementation studies of legal interventions commissioned by gambling regulators in various countries. In addition, we will supplement with searches of books and book chapters, and conference proceedings, to ensure inclusion of qualitative case studies.^56^,^58^

### Search strategy

An information specialist (JK) will support the searches. The search strategy will be informed using keywords reflecting the concepts listed in the PICOST (see table 1) described above. Databases will be searched alongside hand searches of citations, grey literature, books/chapters and conference proceedings. To account for the interdisciplinary nature of this review, inclusive search terms will be used to capture all eligible interventions.

We will undertake searches from inception to 31 August 2024, without limitation on date, language or publication type. Translation will be supported by native language speakers in the Institute of Applied Health Research or Birmingham Law School. Where unavailable, Google Translate will support title and abstract screening. If included for full text screening, funds are allocated to support translation. The search will contain terms relating to the following four themes:

1. Gambling (eg, gambl*or betting or bet(s) or casino or bookmaker).
2. Legal (eg, legislat* or statutory or legal* or law or laws or mandat* or ban or banning).
3. Public health (eg, policy or policies or intervention* or program*or measures).
4. Specific public-health focused consumer protection measures or safeguards (eg, *social responsibility or *reminder systems).

The search strategy will include both free-text terms (ie, searches in the title and abstract) and subject headings (eg, MeSH in MEDLINE). For an example of the search strategy, please see table 2.

**Table 2:** Medline search strategy

#	Query	Results from 27 August 2024
1	GAMBLING/	7236
2	gambling.ti,ab,kw.	10 030
3	(gambler or gamblers).ti,ab.	2907
4	(gamble not standard gamble).ti,ab,kw.	1473
5	(betting or bettor* or wager*).ti,ab.	1155
6	((bets or bet) not (“bet hedging” or hedge* or inhibitor* or inhibition or T Bet or proteins)).ti.	1782
7	(bingo or lottery or lotteries).ti.	674
8	((gaming or slot or fruit or poker or lottery or lotteries) adj5 (machine* or terminal*)).ti,ab.	930
9	((game or games or gaming or gamer?) adj5 (money or monetization or monetisation or monetary)).ti,ab.	260
10	(lootbox or loot box or lootcrate or loot crate or game credit or microtransaction or in game purchase).ti,ab.	53
11	(Casino* or cashino* or bookmaker* or book maker* or bookie* or amusement arcade*).ti,ab.	764
12	1 or 2 or 3 or 4 or 5 or 6 or 7 or 8 or 9 or 10 or 11	15 431
13	Gambling/pc(Prevention & Control)	226
14	Legislation as Topic/	16 025
15	Legislation & Jurisprudence.fs.	262 722
16	Licensure/	7557
17	Government Regulation/	21 953
18	(legislat* or statutory or legal* or law or laws or mandat* or ban or banning or bans or prohibit* or reform* or prohibit* or licences or licencing or licenced or jurisprudence or ordinance or forbid* or interdict*).ti,ab.	469 980
19	(act or acts or restrict* or code or requirement).ti.	128 438
20	((regulation* or regulatory or regulate*) not (emotion* adj2 regul*)).ti.	516 133
21	(statutory adj2 (code* or regulation*)).ab.	219
22	(regulatory adj2 (authorit* or approach* or change* or reform*)).ti,ab.	10 069
23	(gambling adj2 (board* or regulat* or commission* or authorit*)).ti,ab.	120
24	(Lawmaker* or law maker* or government* or police).ti,ab.	176 455
25	(Compliant or compliance or enforce* or sanction* or prosecution* or deregulation).ti,ab.	229 725
26	14 or 15 or 16 or 17 or 18 or 19 or 20 or 21 or 22 or 23 or 24 or 25	1 619 960
27	*Public Health/	58 511
28	*Harm reduction/	2030
29	exp *Government/	46 144
30	exp *policy/	106 837
31	(Harm* adj2 (consumer* or minimi?* or prevent* or reduc*)).ti,ab,kw.	17 925
32	(health adj2 (ministr* or department*)).ti,ab.	51 768
33	“public health”.ti,ab,kw.	386 723
34	(policy or policies or intervention* or program* or measures or postpolicy or prepolicy or policymaker* or policy maker*).ti,kw.	628 523
35	((debt* or relationship* or crime*) adj3 (intervention* or program*)).ab.	3635
36	27 or 28 or 29 or 30 or 31 or 32 or 33 or 34 or 35	1 156 325
37	*Social Responsibility/	8134
38	*Reminder Systems/	2315
39	(Reduction adj1 (demand or supply or opportunity or access)).ti,ab.	462
40	consumer protection.ti,ab,kw.	925
41	(Protect* adj2 (play* or behavio?r)).ti,ab.	10 687
42	(responsib* adj1 social*).ti,kw.	1266
43	((stake* or spending or loss or price or monetary or time or deposit) adj2 (limit* or maximum or capping or restriction)).ti,ab.	33 284
44	Persuasive design.ti,ab.	50
45	(Messag* or warning or pause* or break*).ti.	73 592
46	((messag* or banner) adj1 (static or dynamic or pop-up or safer or warning or health or intervention or responsib*)).ti,ab.	5497
47	(Advertising or marketing or sponsorship*).ti.	14 955
48	(watershed or pre-watershed).ti,ab.	12 359
49	(feedback adj1 (behavio?r or personalis?*)).ti,ab.	241
50	(risk rating or play tracking or play scan or playscan or cashless or card based or acceptor* or pre commitment or precommitment or affordability check* or self exclusion or self appraisal or break play).ti,ab.	74 522
51	(age adj1 (limit* or legal or minimum)).ti,ab.	4577
52	(limit or limits or limiting).ti.	45 715
53	37 or 38 or 39 or 40 or 41 or 42 or 43 or 44 or 45 or 46 or 47 or 48 or 49 or 50 or 51 or 52	283 811
54	26 or 36 or 53	2 848 828
55	12 and 54	2548
56	13 or 55	2625

### Study records

#### Data management

The study will be reported in line with PRISMA—Equity (PRISMA—Equity)^59^ and PRISMA—Harms (PRISMA—Harms).^60^ A flow chart tracking the PRISMA^61^ statement will be developed to detail the data collection, selection and extraction process. Search results will be managed using Covidence software.^62^

#### Data selection and collection process

Using Covidence,^62^ two independent reviewers (MF and TP) will remove duplicate items and screen titles/abstracts against the inclusion criteria (KB, GJM-T and PM will resolve conflicts). Full texts that meet the inclusion criteria will be screened by the same reviewers (MF and TP) (KB, GJM-T and PM will resolve conflicts). Data will be extracted to a form, developed by KB, GJM-T and PM, also using the Covidence software.^62^

### Data items

Extracted variables will include:

1. Author and year.
2. Study design.
3. Population and setting (N, age range of participants, sociodemographics, location).
4. Intervention details (name, description of intervention, content of intervention).
5. Effect size (eg, measured outcomes from validated assessment scales such as Problem Gambling Severity Index score and/or reported effect on any other gambling-related harm variables (detailed in the PICOST)), grouped by PROGRESS-Plus criteria^63^ where applicable, for example, just in women, just in minority groups, just in areas of deprivation.
6. Quality assessment.

### Outcomes and prioritisation

The primary outcome is to ascertain the effects of interventional PH laws and regulations on gambling-related harms. Main outcomes will include all changes in gambling-related harms measures including financial harm, crime, societal harm, familial harm and self-reported measures (eg, a reduction in the reported Problem Gambling Severity Index score). Secondary outcomes will include but are not limited to (1) the effectiveness of interventions at compliance and enforcement level, (2) the cost-effectiveness of interventions and (3) any other positive, negative and spillover effects (including economic costs, paradoxical effects and harmful externalities, including widened health inequalities).

### Risk of bias in individual studies

The risk of bias and quality of the included studies will be assessed using different tools for different types of study. Randomised controlled trials will be assessed using the Cochrane risk of bias tool (RoB2).^64^ Risk Of Bias In Non-randomised Studies - of Interventions (ROBINS-I)^65^ will be used for other non-randomised studies. Qualitative studies will be appraised using the EPPI reviewer software for systematic reviewing.^66^ Two independent reviewers will perform the assessment (MF and TP), with any conflicts adjudicated by a third reviewer (KB, GJM-T or PM). Where possible, the quality will be scored (inadequate, adequate, good, excellent or unclear). Inclusion will not be determined by quality rating, due to anticipated heterogeneity between studies and the planned comparison between industry and non-industry funded studies.

### Data synthesis

Included studies will be heterogeneous. We will conduct a narrative synthesis to interpret the data. Narrative synthesis will be guided by existing literature that categorises the effects (including unintended and spillover effects (eg, the INTENTS framework^67^) of interventional PH laws. Any additional categories reported in the studies we encounter will also be considered. It is anticipated that studies will be synthesised by type of law/regulation (eg, a universal prohibition; a targeted prohibition); key outcomes; types of unintended effect; and/or subgroups impacted.

We will undertake an iterative process of developing CMOCs to explain the effects we uncover and define a theory of change. Preliminary CMOCs (see figure 1) will be refined further after the narrative synthesis stage of the review. In addition, we will identify demiregularities linked to specific explanatory factors^68^ after broadening our analysis to generate new questions and fields of discovery.^69 70^ Analysis will stop when no new configurations can be developed and supported based on the collective evidence. CMOCs will be labelled with respect to the strength and diversity of the evidence supporting them.

We are aware that industry-funded studies—in gambling and other sectors—routinely suggest that interventional PH laws and regulations impose fiscal harms in terms of lost revenue and impose costs on businesses and consumers.^71^ Without effective segmentation of industry-funded research, there is a risk that reviews of evidence may be skewed to a proindustry, antiregulation perspective focused on outcomes for businesses, rather than health and health equity outcomes.^72^ We will segment our search results and narrative synthesis such that industry-funded/conflicted research on intervention effects can be considered separate from the main pool. This will enable a substudy of how industry funding of studies impacts findings on the effects of interventional PH laws and regulations oriented to reducing gambling harm.

## Ethics and dissemination

Our review is registered with PROSPERO (International prospective register of systematic reviews) at the National Institute for Health Research and the Centre for Reviews and Dissemination (CRD) at the University of York (CRD42024574502) and will follow the Realist And Meta-narrative Evidence Syntheses: Evolving Standards (RAMESES) reporting guidance for realist reviews.^73^

We aim to define a theory of change and produce a context-mechanism-outcome framework with relevant experts using the findings. We plan to disseminate the findings through peer-reviewed publications, meetings with relevant experts, international conference presentations, a Delphi consensus study (workshops and/or focus groups).

### Coproducing an analytical framework

The development of CMOCs will include a collaborative exercise with relevant experts and PPI members recruited from our network of healthcare, charity and community stakeholder connections. Following the data synthesis, the findings will be shared with a group of relevant experts (n=15). These will include experts by experience in gambling harm, gambling researchers, charities, service providers and regulators. Alongside the research team, and using a focus group format, these experts will coproduce a revised analytical framework. The outcome will be the second iteration of CMOCs (see section 1.1.1 for the first iteration). The revised analytical framework will also assist the research team to negate meta-biases and confidence in the cumulative evidence by enabling an additional level of expert appraisal of the systematic review evidence.

### Meta-bias

The evidence will be carefully extracted and scrutinised by an interdisciplinary team of law and applied health research specialists (KB, MF and TP). Any disagreements in screening, extraction and appraisal will be presented to the wider research team for discussion (JSC, SM, GJM-T and PM), with decisions reached by consensus. A subsequent coproduction activity will assist in addressing researcher bias by incorporating expert opinion to the evidence synthesis during CMOC development and subsequent definitions of theory of change.

In addition, to address bias and improve awareness from a health equity perspective, we plan to formally integrate an equity lens into the systematic review process by utilising the FOR EQUITY tool.^74^ FOR EQUITY was developed by NIHR as a toolkit to integrate ‘intersectional health inequity' in health research. FOR EQUITY assists the research team to critically and conceptually understand the drivers of inequalities relevant to this review. Conceptualisation of the determining factors underpinning health inequities is achieved using a three-step approach of ‘REFLECT, INVOLVE and DIVE DEEPER’, alongside structured reflections on responsibilities of the research team and research institution, to involve under-represented groups in analysis.

### Confidence in cumulative evidence

We will follow the RAMESES reporting guidance for realist reviews.^73^ Applying the context of realist theory means outcomes are considered conditionally to the circumstances in which they were reported. We will also consult the Grading of Recommendations Assessment, Development and Evaluation guidelines^75^ to test that our overall recommendations are of a high quality.

Evidence will reflect a real-world context, with our narrative synthesis enhanced by contributions from relevant experts through the coproduction of an analytical framework (CMOCs) and definition of theory of change. Once we have gathered evidence from the realist review of legal PH interventions on what works, why, for whom, in what context, and on unintended effects and mechanisms for actions, we will develop an explanatory account of the effects of gambling law and regulation on gambling harm.
