# Supplementary material for: Effects of interventional public health laws and regulations intended to reduce gambling-related harms: a realist review study protocol
Source: BMJ Open. 2025 Jul 16;15(7):e093906. doi: 10.1136/bmjopen-2024-093906 (PMC12273126; doi:10.1136/bmjopen-2024-093906)
Supplement: Supplementary file 1 [file bmjopen-2024-093906.reviewer_comments.pdf]

PEER REVIEW HISTORY

BMJ Open publishes all reviews undertaken for accepted manuscripts. Reviewers are asked to complete a checklist review form and are provided with free text boxes to elaborate on their assessment. These free text comments are reproduced below.

ARTICLE DETAILS

Title (Provisional)

Effects of interventional public health laws and regulations intended to reduce gambling related harms. A realist review study protocol

Authors

Fisher, Michelle; Piper, Theodore; Mavi, Sonia; Nambiar, Jaya; Sharma, Priyanka Kumar; Kirby, Joelle; Melendez-Torres, G.J; Montgomery, Paul; Fewell, Graham; Chandan, Joht Singh; Bedford, Kate

---

VERSION 1 - REVIEW

Reviewer 1

Name Rintoul, Angela

Affiliation Federation University Australia, Health Innovation and Transformation Centre

Date 08-Nov-2024

COI None

This review protocol promises to provide valuable evidence and outlines a process that should fill important gaps in knowledge. My main question relates to the scope of the review for instance one key intervention to protect public health would (arguably) be to prohibit particular/all forms of gambling. Albeit few jurisdictions continue to take this approach, it presumably would remain one of the major instruments of 'intervention'? Aside from this, I have only a few other comments.

While recognising the teams interdisciplinary approach, have the authors considered the utility of contextualising this research in terms of legal epidemiology? Some additional resources or references or relevance may for instance be: https://www.annualreviews.org/content/journals/10.1146/annurev-publhealth-032315-021841

The authors note that PPI involvement helped with the design of the conceptual model. Who are the 'patients and public' in this? Some more details about this would be useful to include in the methods, ethics, recruitment, timing etc.

Search terms line 234 –in some jurisdictions (i.e. Australia, and NSW in particular) the term gaming refers to electronic gambling machines, and the team may wish to consider adding *gam to their list of terms.

It's good to see that the authors are intending to use a far wider set of potential sources of evidence, but for instance I don't think the CDC community planning tool contains any information relating to gambling? If my understanding is correct, I'm not sure there is value in listing this as a source of information for the review?

I look forward to reading the results of this work.

VERSION 1 - AUTHOR RESPONSE

Reviewer	Recommendation	Response	Location
Reviewer 1	Patient and Public Involvement statement with its own subheading in the Methods	Section 2.1 refers to the patient and public involvement and explains what PPI activity was undertaken for this protocol	See lines 161 - 165
Reviewer 1	One key intervention to protect public health would (arguably) be to prohibit particular/all forms of gambling. Albeit few jurisdictions continue to take this approach, it presumably would remain one of the major instruments of 'intervention'.	We have included prohibition in our criteria of intervention as it fits the criteria of legal regulation. As such we have added prohibition as an example in the types of regulations listed in the introduction to be consistent with what is described later in the data synthesis section.	See line 68 & 300
Reviewer 1	While recognising the teams interdisciplinary approach, have the authors considered the utility of contextualising this research in terms of legal epidemiology?	Thank you for highlighting this. The review is based upon a legal epidemiological context. We have added the term and further justification to the introduction to be more direct on our stance.	See lines 71, 82 & 99
Reviewer 1	Search terms line 234 –in some jurisdictions (i.e. Australia, and NSW in particular) the term gaming refers to electronic gambling machines, and the team may wish to consider adding *gam to their list of terms.	This point was considered during the development of the review. However, adding the term gam* generated an unmanageable amount of search results. The search has now been executed and cannot be adapted at this stage, however, we aim to describe and address this in our limitations.	NA
Reviewer 1	It's good to see that the authors are intending to use a far wider set of potential sources of evidence, but for instance I don't think the CDC community planning tool contains any	We have identified some reference to commercial gaming venues which may have been relevant.	NA

	information relating to gambling? If my understanding is correct, I'm not sure there is value in listing this as a source of information for the review?		
